# Design science research in quality improvement: Embedding rigour in digital health innovation

**DOI:** 10.4102/phcfm.v17i2.5194

**Published:** 2025-11-30

**Authors:** Robin E. Dyers, Hassan Mahomed, Darelle van Greunen

**Affiliations:** 1 Division of Health Systems and Public Health, Department of Global Health, Faculty of Medicine and Health Sciences, Stellenbosch University, Cape Town, South Africa; 2 WHO-FIC Collaborating Centre, Burden of Disease Research Unit, Cape Town, South Africa; 3 Department of Health and Wellness, Western Cape Government, Cape Town, South Africa; 4 Centre for Community Technologies, Faculty of Engineering, The Built Environment and Technology, Nelson Mandela University, Gqeberha, South Africa

**Keywords:** design science research; quality improvement, digital health, healthcare innovation, primary health care

## Abstract

Design science research (DSR) transforms how healthcare researchers create digital innovations by treating artefacts as knowledge repositories rather than mere technical solutions. It provides a problem-solving paradigm that creates artefacts embodying prescriptive knowledge about solving classes of problems, complementing quality improvement methodologies. Through its systematic approach, DSR equips healthcare researchers with methods for building digital health innovations, using quality improvement concepts as reference points to facilitate understanding and adoption. The methodology presents philosophical foundations distinguishing design sciences from natural sciences, five artefact types (constructs, models, methods, instantiations, design theories), and a six-phase framework (problem identification, objectives, design, demonstration, evaluation, communication). Systematic problem investigation transforms vague complaints into measurable problems amenable to designed solutions. This paradigm distinguishes itself as one where the artefact is the knowledge contributor. While quality improvement produces innovations solving problems, DSR produces artefacts embodying prescriptive knowledge about solving classes of problems. Both methodologies innovate; the distinction lies in knowledge representation. The DSR approach treats artefacts as knowledge repositories containing extractable design principles, while quality improvement focuses on demonstrating improved outcomes. Methodological synergies strengthen both approaches through complementary evaluation frameworks and iterative refinement. Practical considerations include maintaining methodological rigour through transparent documentation, addressing AI integration challenges, ensuring sustainability, and avoiding common pitfalls. African healthcare contexts particularly benefit from DSR’s orientation, with resource constraints demanding solutions addressing complex socio-technical challenges while contributing to global design knowledge. Future research should establish DSR training programmes and develop artefact repositories for systematic knowledge transfer, positioning African researchers as contributors to healthcare’s digital transformation.

## Introduction

Healthcare systems worldwide face complex problems demanding innovative solutions that improve patient outcomes while navigating resource constraints. Design science research (DSR) emerges as a problem-solving paradigm that fundamentally reorients research from understanding what exists to creating what should exist, providing rigorous methods for developing innovative healthcare solutions.^1,2^

Many digital health interventions emerge as solutions in search of problems, developed without systematic problem identification or rigorous evaluation of their efficacy in addressing genuine healthcare challenges. The proliferation of coronavirus disease 2019 (COVID-19) contact tracing applications illustrates this methodological gap: despite technical sophistication, many apps were developed without clear problem definition, user needs assessment, or evaluation frameworks to determine whether they achieved intended outcomes.^3,4^ While numerous digital health tools emerged during the pandemic across African countries, most lacked systematic evaluation of their actual contribution to pandemic response.^5^ This pattern, where technical capability drives development rather than identified needs, signals the absence of systematic approaches to artefact creation and evaluation.

Quality improvement (QI) has established itself through approaches including Plan-Do-Study-Act (PDSA) cycles, Lean methods, Six Sigma and the Model for Improvement, demonstrating success in enhancing care delivery processes.^6^ However, these methodologies primarily address improving existing processes rather than creating fundamentally new solutions.^7^

Design science research offers a complementary paradigm specifically designed for creating and evaluating novel artefacts. Unlike descriptive research studying existing phenomena, DSR generates knowledge through building solutions.^2^ Experimental research tests existing interventions; DSR creates new ones. Both approaches complement each other, with descriptive methods informing problem identification and experimental designs evaluating created artefacts.^8^ This constructive orientation proves particularly valuable for healthcare contexts characterised by complex socio-technical interactions.

African primary health care contexts present particularly complex challenges that DSR uniquely addresses. Resource constraints and diverse cultural contexts demand simultaneously innovative and pragmatic solutions. While sub-Saharan African countries are increasingly adopting digital health technologies to improve health financing and universal health coverage,^9^ most pilots remain unsustainable because of external funding dependencies and methodological limitations.^10^ However, successful DSR applications are emerging in African healthcare contexts, with South African medical radiation science education demonstrating how systematic DSR methodology can develop and evaluate educational artefacts that achieve sustainable innovation.^11,12^

## Understanding design science research

### Core principles

Design science research operationalises systematic artefact creation through philosophical foundations rooted in Herbert Simon’s seminal work, ‘The Sciences of the Artificial’, which distinguishes between studying natural phenomena and studying human-designed constructs.^13^ Unlike natural sciences discovering existing phenomena, DSR embraces constructive activities as legitimate knowledge-building processes through five interconnected principles.

- *Problem-oriented nature* positions DSR as beginning with identifying meaningful problems in practice rather than theoretical abstractions.^1^ Healthcare contexts exemplify this orientation, where complex socio-technical challenges demand solutions addressing real-world constraints and stakeholder needs.^14^
- *Artefact-centredness* establishes that knowledge creation occurs through designing and building artefacts. In DSR, an artefact is any designed solution addressing a problem, not exclusively digital technologies.^1^ These include: constructs (vocabularies, frameworks), models (representations of problems and/or solutions), methods (algorithms, clinical protocols), instantiations (working implementations proving feasibility), and design theories (prescriptive knowledge for solution classes).^15^ Examples span from triage frameworks and patient flow diagrams to functioning appointment systems. These artefacts serve as both research outputs and knowledge vehicles, embedding transferable design knowledge.
- *Dual contribution* ensures DSR generates both practical solutions and reusable design knowledge.^2^ While artefacts provide immediate utility within specific contexts, the design knowledge embedded within successful artefacts becomes transferable across similar problem domains, distinguishing DSR from pure development work.^16^
- *Evaluation-driven validation* demands that artefacts demonstrate utility and efficacy through systematic assessment.^2^ Efficacy refers to performance under controlled conditions, while effectiveness refers to real-world performance; DSR evaluation encompasses both through Venable et al.’s framework.^8^
- *Iterative refinement* recognises that learning occurs through build-evaluate cycles that progressively improve artefact performance and deepen understanding.^2,7^ This iterative approach proves particularly valuable for healthcare contexts where stakeholder requirements emerge through use rather than specification alone.

These principles collectively ensure scientific rigour while delivering practical value, proving particularly relevant for healthcare innovation where solutions must satisfy both theoretical contribution requirements and clinical utility demands.

### Artefact types

March and Smith identified four fundamental artefact types, with design theories added as a fifth type representing abstract design knowledge^1,17^:

- *Constructs* form foundational vocabulary for problem and solution spaces, including frameworks for patient engagement or digital health readiness taxonomies.
- *Models* represent relationships between constructs, depicting clinical information flows or intervention-outcome relationships.
- *Methods* provide systematic procedures through defined steps and decision criteria, including digital health assessment protocols or clinical decision algorithms.
- *Instantiations* demonstrate feasibility through working implementations, from prototype applications to deployed clinical systems. Unlike the other artefact types which remain conceptual (vocabularies, model diagrams, methods), an instantiation is the actual functioning system. For example, while a method might describe the steps for triaging patients, the instantiation would be the actual working triage software installed in the emergency department that staff use daily.
- *Design theories* synthesise learning into generalisable principles through eight components including purpose, constructs, principles, and testable propositions.

### Design science research process framework

**FIGURE 1:**
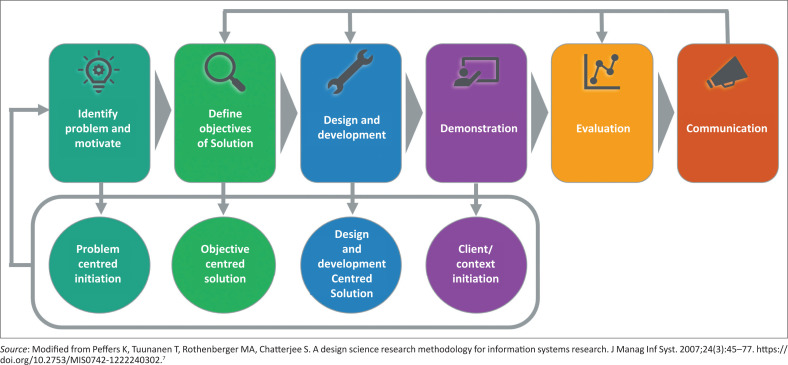
Design science research process.

Peffers et al.’s six-phase methodology structures DSR projects whilst accommodating diverse entry points (Figure 1).^7^ While traditional healthcare intervention development focuses on creating and testing solutions, DSR uniquely requires that the solution itself embodies transferable design knowledge. Each design decision must be explicitly documented to enable others to apply the principles to similar problems. The distinction is that DSR doesn’t just ask ‘Does it work?’ but also ‘What can others learn from how we built it?’:

- *Problem identification* establishes foundations by articulating innovation needs through deep healthcare context engagement.
- *Solution objectives* transform problem understanding into measurable design goals balancing ambition with feasibility.
- *Design and development* combine knowledge and imagination to produce novel solutions through iterative prototyping and stakeholder feedback.
- *Demonstration* provides initial viability evidence through application in authentic healthcare environments, moving from controlled to naturalistic settings.
- *Evaluation* rigorously assesses whether solutions achieve objectives. The Framework for Evaluation in Design Science (FEDS) guides evaluation through two dimensions: formative versus summative evaluation (when to evaluate) and artificial versus naturalistic settings (where to evaluate).^8^
- *Communication* disseminates learning through academic publications and practical implementation guidance, ensuring innovations achieve impact beyond individual projects.

These phases form iterative cycles rather than linear sequences, essential for healthcare innovation where complex requirements rarely yield to single-pass solutions.^15^

## Application framework for digital health

### Innovation

#### Problem identification

Problem identification in DSR requires systematic investigation beyond surface symptoms.^7^ Rather than accepting problem statements at face value, researchers must establish empirical baselines and measurable problem dimensions.

The ePharmacare project (Box 1) exemplifies this through time-motion studies revealing pharmacists spent only 50% of time on patient care.^18^ This quantification transforms vague complaints about ‘lack of time’ into specific, measurable problems. Design science research demands such precision to enable later evaluation of whether solutions address identified problems.

BOX 1 Complete DSRM implementation of ePharmacare.

**ePharmacare demonstrates the six phases of DSRM in action:**

**Problem:** Pharmacists spend only 50% of time on patient care because of administrative burden.

**Objectives:** Enable pharmaceutical care without increasing workload through an online platform.

**Design:** ePharmacare platform (instantiation) for remote monitoring, medication management, refill alerts.

**Demonstration:** Field tested with 28 chronic patients in pharmacy settings.

**Evaluation:** Tasks completable within available time; identified adoption prerequisites (IST skills, training needs).

**Communication:** Disseminated throughout project via conferences, practitioner engagement, and peerreviewed publications.

**Key insight:** Illustrates DSR’s iterative nature where evaluation informs subsequent design cycles.

DSRM, Design Science Research Methodology; DSR’s, Design Science Research; IST, Information Systems and Technologies.

#### Objectives

Objectives translate problems into solution criteria, establishing what success looks like before designing anything.^7^ This prevents solution-driven thinking where available technology seeks problems to solve.

DSR objectives serve dual purposes: guiding practical solution development while enabling theoretical contribution.^19^ ePharmacare’s objectives demonstrate this duality – ‘enable pharmaceutical care without increasing workload’ addresses the immediate 50% time constraint while testing whether online platforms can shift routine tasks without compromising care quality, a transferable design principle.^18^

Setting objectives before selecting solutions is critical. Had ePharmacare started with ‘implement an online platform’, the project would have missed exploring alternative solutions. Instead, workload-neutral pharmaceutical care as the objective allowed various solution pathways.

#### Design

Design transforms objectives into concrete artefact specifications through systematic creativity.^7^ Unlike pure creative design, DSR employs established theories and proven design principles to guide solution development within scientific constraints.^17^

The design phase requires balancing competing demands: theoretical rigour versus practical constraints, innovation versus feasibility, comprehensiveness versus usability. ePharmacare’s design decisions illustrate these trade-offs.^18^ Web-based architecture balanced accessibility against functionality. Task flows accommodating 7 min – 8 min completion windows addressed fragmented time availability. Bidirectional data entry (pharmacist and patient) distributed workload while maintaining clinical oversight.

Critical to DSR design is maintaining traceability from objectives to design choices. Each ePharmacare feature was mapped to identified objectives: medication tracking addressed adherence monitoring; refill calculations reduced administrative burden; asynchronous messaging fitted micro-break workflows. This systematic linkage distinguishes DSR from ad hoc development.^1^

Iterative refinement through user feedback ensures designs remain grounded in practical reality while pursuing innovation.^2^

#### Demonstration

Demonstration tests whether artefacts function in intended environments, providing proof-of-concept before comprehensive evaluation.^7^ This phase answers ‘Can it work?’ rather than ‘How well does it work?’ – establishing feasibility before effectiveness assessment.^2^

ePharmacare’s demonstration deployed the platform in pharmacy settings, confirming core functionality: pharmacists completed tasks, patients entered data, systems synchronised correctly.^18^ However, demonstration revealed implementation prerequisites invisible during design: recruitment protocols, training requirements, and engagement structures. This distinction between technical functionality and practical feasibility characterises effective demonstration.^8^

For digital health, demonstration in authentic clinical environments with actual users provides early warning of adoption barriers, enabling iteration before costly evaluation. Projects skipping demonstration risk discovering fundamental feasibility issues only during expensive evaluation phases, a common cause of digital health implementation failure.^20^

#### Evaluation

Evaluation assesses the extent to which artefacts solve the problem identified in phase one, closing the DSR loop.^8^ This phase must answer: ‘Did we solve the original problem?’ not merely ‘Does the artefact work?’

ePharmacare measured task completion times (7 min 38 s, fitting within available windows) and usability metrics, but incompletely assessed whether the platform increased pharmacists’ patient care time from 50%.^18^ This gap illustrates a common DSR pitfall: evaluating technical success rather than problem resolution.

Rigorous DSR evaluation requires returning to the original problem metrics. If the problem was ‘50% patient interaction time’, the evaluation must measure whether this percentage improved. Without this alignment, projects risk declaring success based on functional artefacts that fail to address identified problems. This disciplined focus on problem-solution fit distinguishes DSR from pure development work.

#### Communication

Communication disseminates both artefact details and design knowledge, enabling others to adapt solutions to their contexts.^19^ ePharmacare’s multichannel dissemination (conferences, journals, practitioner engagement) exemplifies comprehensive communication.^18^ Design science research requires sharing not just what was built but why design decisions were made, enabling knowledge transfer beyond specific instances.

## Design science research and quality improvement synergies

While the ePharmacare project demonstrates DSR’s systematic approach to creating digital health innovations, healthcare professionals may recognise parallels with quality improvement methodologies already embedded in their practice. Rather than competing approaches, DSR and quality improvement reveal complementary strengths that, when understood together, help researchers new to DSR recognise familiar concepts from their QI experience.

### Methodological parallels

Where DSR creates novel artefacts through systematic design principles, quality improvement ensures effective integration within healthcare workflows through iterative refinement.^21^ This complementarity addresses the implementation gap where healthcare innovations, including digital tools, clinical protocols and organisational structures, fail despite technical sophistication.

DSR’s rigorous evaluation frameworks parallel quality improvement’s organisational readiness assessments. Quality improvement brings practical process measurement, while DSR contributes systematic design documentation. For instance, ePharmacare’s time-motion studies (a QI technique) provided the empirical foundation for DSR’s problem identification phase. Recent systematic reviews confirm that methodologies sharing these characteristics achieve superior outcomes.^21^

### Iterative development similarities

Both methodologies use cycles, creating natural conceptual bridges for QI-trained researchers. Plan-Do-Study-Act’s rapid testing resembles DSR’s iterative refinement within its broader framework. Consider how ePharmacare’s demonstration phase mirrors PDSA’s ‘Do’ phase. Both test solutions in real settings. The distinction lies in DSR’s emphasis on extracting transferable design knowledge, not just local optimisation.

### Evaluation approach commonalities

Design science research’s FEDS framework shares QI’s commitment to rigorous assessment.^6,8^ Quality improvement’s process, outcome and balancing measures resemble DSR’s multifaceted evaluation criteria. Both ensure that solutions address the original problems, not just function technically. Recognising these parallels helps QI-experienced researchers apply familiar evaluation thinking to DSR projects.

### Mapping familiar concepts

The parallels between DSR phases and quality improvement concepts become clearer when mapped directly. Table 1 illustrates how each DSR phase corresponds to familiar QI approaches, helping researchers transition between methodologies. These mappings are not prescriptive equivalences but rather conceptual bridges that facilitate understanding. For instance, while root cause analysis serves problem identification in QI, DSR extends this through systematic stakeholder analysis and empirical baseline establishment.

**TABLE 1:** Mapping design science research phases to familiar quality improvement concepts.

DSR phase	Familiar QI concept	Shared purpose
Problem identification	Root cause analysis	Evidence-based problem definition
Objectives	SMART goals, aim statements	Measurable success criteria
Design	Improvement ideas, workflow redesign	Systematic solution development
Demonstration	PDSA ‘Do’ phase	Real-world testing
Evaluation	QI metrics framework	Performance assessment
Communication	Best practice sharing, spread strategies	Knowledge dissemination

QI, quality improvement; PDSA, Plan-Do-Study-Act; DSR, design science research.

Understanding these parallels helps healthcare researchers recognise that DSR builds upon familiar QI foundations whilst adding systematic artefact creation and design knowledge generation. African healthcare contexts benefit from researchers who understand both approaches, applying DSR’s innovation capacity with QI’s implementation wisdom.

## Practical considerations

### Methodological rigour

Design science research requires systematic documentation enabling others to understand and evaluate design decisions.^2^ This rigour distinguishes research from routine system development.

Practical rigour means maintaining clear records throughout the project:

#### Problem evidence

Document how problems were identified and measured (time studies, surveys, observation data).

#### Design rationale

Record why specific design choices were made (platform selection, interface decisions, functionality priorities).

#### Evaluation alignment

Ensure evaluation metrics connect to original problems (if the problem was time allocation, measure time changes).

Rigour does not require complex validity frameworks or theoretical perfection. It means being transparent about methods, maintaining decision trails, and honestly reporting both successes and failures. When projects reveal unexpected barriers or fail to achieve objectives, documenting these findings contributes valuable knowledge. This transparency enables others to learn from the work, avoiding repeated mistakes and building on partial successes.

Healthcare professionals implementing DSR need not become methodology experts but should maintain sufficient documentation for others to understand what was undertaken, why it was undertaken, and what was learned.

### Artificial intelligence and machine learning integration

Artificial intelligence (AI) technologies increasingly shape healthcare DSR, offering capabilities for pattern recognition and decision support. Successful integration requires addressing algorithmic transparency, with healthcare demanding explainable AI enabling clinical understanding of recommendations.^22^ Data representativeness poses particular challenges, requiring local training data and bias detection across demographic subgroups.

### Ethical and sustainability considerations

Healthcare DSR must balance immediate patient needs with environmental sustainability. Technical sustainability requires technologies maintainable with local expertise, avoiding external dependency. Organisational sustainability demands embedding innovations within existing structures. Justice considerations require explicit attention to equity throughout design, ensuring accessibility for marginalised populations.

### Common pitfalls

Several recurring challenges threaten project success globally. Misalignment between design features and user needs occurs when developers prioritise technical novelty over practical value. To mitigate this misalignment, projects require continuous stakeholder engagement, iterative prototyping, and explicit documentation of design decisions grounded in user requirements rather than technical possibilities.

Even innovations that succeed technically can fail if active change management is ignored. Healthcare professionals may resist technologies disrupting workflows or challenging autonomy. Successful projects invest in training, provide ongoing support and demonstrate clear value propositions. Hevner’s relevance cycle emphasises continuous stakeholder engagement throughout the design process, ensuring solutions address genuine user needs while building confidence through iterative demonstration and feedback.^2^

Poor knowledge management limits cumulative learning when researchers fail to document design rationale, implementation challenges or contextual factors. Establishing repositories and publishing successes and failures strengthens the DSR community. Research within African healthcare settings contributes valuable insights by documenting innovative adaptations that achieve excellence within resource parameters.

External dependency undermines sustainability when projects rely on international funding or imported technologies without building local capacity. Healthcare systems across Africa increasingly prioritise technological sovereignty, developing home-grown solutions that leverage local expertise. Successful strategies include capacity building since inception, establishing local maintenance capabilities, and designing systems that remain under local control while fostering indigenous technological advancement.

## Conclusion

This article has introduced design science research as a problem-solving methodology for creating and evaluating health innovations in African primary health care contexts, demonstrating how systematic artefact development complements existing quality improvement approaches.

Design science research distinguishes itself as a paradigm where the artefact is the knowledge contribution. Quality improvement produces innovations that solve problems; DSR produces artefacts that embody prescriptive knowledge about how to solve classes of problems. In DSR, the act of building generates theory – the artefact contains design principles that can be extracted, studied, and instantiated in new contexts. Quality improvement asks ‘does this work better?’; DSR asks ‘what design knowledge does this artefact contain that others can use?’ Both methodologies innovate and can create entirely new paradigms. Both create interventions that solve problems and can operate at local, regional, or system-wide scales. The distinction lies in primary intent and knowledge documentation. Quality Improvement focuses on demonstrating improved outcomes, with design knowledge often remaining tacit within implementation teams. Design science research explicitly treats artefacts as knowledge repositories, systematically documenting design rationale and principles. This makes DSR particularly suited when the goal is research publication and knowledge transfer across contexts, whilst QI excels at operational improvement whether or not formal research dissemination is intended. The distinction lies not in innovation capacity but in knowledge representation: Design science research treats artefacts as knowledge repositories containing extractable design principles, whilst QI focuses on demonstrating improved outcomes.

African healthcare contexts particularly benefit from DSR’s orientation, with resource constraints demanding solutions that address complex socio-technical challenges. Future research should establish DSR training programmes, develop artefact repositories showcasing successful innovations, and create documentation standards for capturing design rationale. These initiatives will advance local innovation capacity while contributing to global design knowledge, positioning African researchers as contributors to healthcare’s digital transformation.
